# Molecular evolutionary and structural analysis of familial exudative vitreoretinopathy associated FZD4 gene

**DOI:** 10.1186/s12862-019-1400-9

**Published:** 2019-03-08

**Authors:** Suman Seemab, Nashaiman Pervaiz, Rabail Zehra, Saneela Anwar, Yiming Bao, Amir Ali Abbasi

**Affiliations:** 1 National Center for Bioinformatics, Program of Comparative and Evolutionary Genomics, Faculty of Biological Sciences, Quaid-i-Azam University, Islamabad, 45320 Pakistan; 2 BIG Data Center & CAS Key Laboratory of Genome Sciences and Information, Beijing Institute of Genomics, Chinese Academy of Sciences, Beijing, 100101 China

**Keywords:** Multigene family, G-protein coupled receptors, Frizzled, FZD4, Metazoans, Phylogenetic analysis, Structural analysis, Familial exudative vitreoretinopathy

## Abstract

**Background:**

Frizzled family members belong to G-protein coupled receptors and encode proteins accountable for cell signal transduction, cell proliferation and cell death. Members of Frizzled receptor family are considered to have critical roles in causing various forms of cancer, cardiac hypertrophy, familial exudative vitreoretinopathy (FEVR) and schizophrenia.

**Results:**

This study investigates the evolutionary and structural aspects of Frizzled receptors, with particular focus on FEVR associated *FZD4* gene. The phylogenetic tree topology suggests the diversification of Frizzled receptors at the root of metazoans history. Moreover, comparative structural data reveals that FEVR associated missense mutations in *FZD4* effect the common protein region (amino acids 495–537) through a well-known phenomenon called epistasis. This critical protein region is present at the carboxyl-terminal domain and encompasses the K-T/S-XXX-W, a PDZ binding motif and S/T-X-V PDZ recognition motif.

**Conclusion:**

Taken together these results demonstrate that during the course of evolution, *FZD4* has acquired new functions or epistasis via complex patter of gene duplications, sequence divergence and conformational remodeling. In particular, amino acids 495–537 at the C-terminus region of FZD4 protein might be crucial in its normal function and/or pathophysiology. This critical region of FZD4 protein may offer opportunities for the development of novel therapeutics approaches for human retinal vascular disease.

**Electronic supplementary material:**

The online version of this article (10.1186/s12862-019-1400-9) contains supplementary material, which is available to authorized users.

## Background

G-protein coupled receptors (GPCRs) are diverse group of membrane proteins, encoded by more than 800 genes in the human genome [1]. GPCRs are found within the plasma membrane and share a common architecture entailing seven-transmembrane (TM) domains [2]. The function of GPCRs is to detect a wide range of extracellular signals, mainly small organic molecules and whole proteins [3]. After detection, GPCRs bind to the ligand and undergo several conformational changes which result in the activation of multiplex signaling networks and a cellular response [4]. GPCRs play a vital role in various physiological processes, ranging from sight, smell and taste to enormous number of neurological, cardio-vascular, and reproductive functions [5]. The involvement of GPCR superfamily in such complex processes makes it a major target for various drug discovery approaches [6]. GPCRs are categorized into five main families namely, glutamate, rhodopsin, secretin, adhesion and Frizzled receptor family [4].

Frizzleds (FZDs) are 7- transmembrane (TM) receptors reside on plasma membrane. They play an important regulatory role in controlling cell polarity and cell proliferation during embryonic development by transmitting signals from glycoproteins, mainly Wnt proteins [7]. Because many Frizzleds have been defined in metazoans, the tissue specific expression of Frizzled genes is a very intricate task [8]. Predominantly, Frizzled receptors are extensively and broadly expressed and almost every single cell expresses one or more Frizzled receptors [9]. The Frizzled family consists of 10 Frizzled receptors, *FZD1, FZD2, FZD3, FZD4, FZD5, FZD6, FZD7, FZD8, FZD9* and *FZD10*. Frizzled receptors vary in length ranging from 537 to 706 amino acids. Exploring differences and similarities among members of Frizzled Receptor family can be helpful in unraveling functional evolution of this family.

Among the members of Frizzled receptor family, *FZD4* is the most finely understood member in context of its biological function, its interaction with ligands and other proteins, and also with respect to its role in human disease phenotype. Several studies suggest that mutations in *FZD4* gene may affect its normal cellular function [10–12]. In humans, a large number of missense mutations in *FZD4* results in a variable amount of retinal hypovascularization, a condition called familial exudative vitreoretinopathy (FEVR) [13]. Mice null for frizzeled4 (*Fzd4*^−/−^) exhibit premature intra-retinal vasculature and thus provide further evidence for a role of FZD4 in retinal angiogenesis [14].

Keeping in view the prominence of Frizzled Receptor family in several developmental processes, the detailed phylogenetic analysis is performed to infer the evolutionary origin and diversification of its members. To investigate the variations in domain topologies, a comparative analysis of known functional domains is conducted. Moreover, due to the substantial role of *FZD4* in FEVR, 40 missense mutations are targeted for comparative structural analysis to scrutinize their impact on overall protein conformation.

## Results

### Phylogenetic analysis

**Fig. 1:**
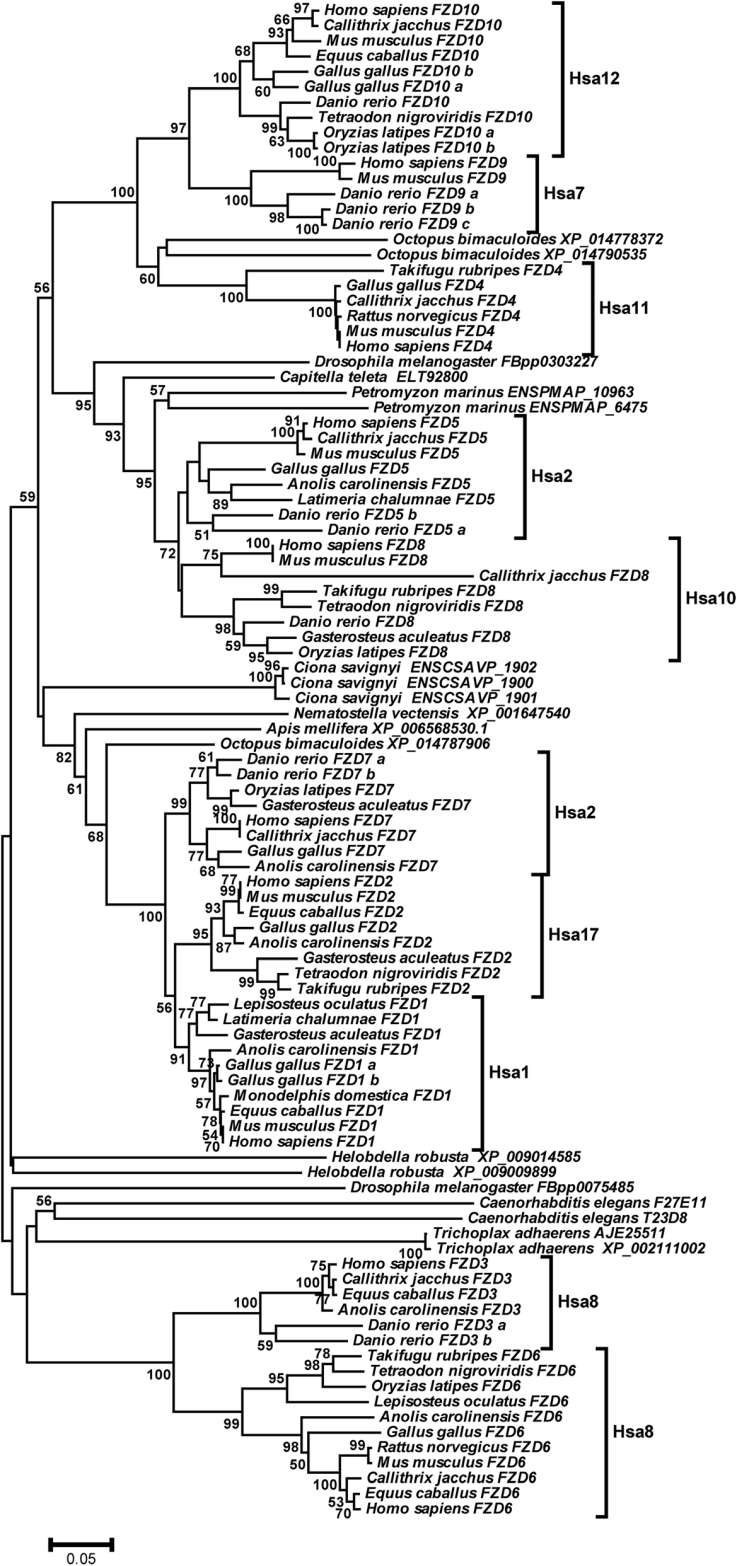
Phylogenetic tree of Frizzled receptor gene family. Evolutionary history of Frizzled receptor gene family was inferred by neighbor joining method. Complete-deletion option was used to eradicate gaps and missing data. Numbers on branches represent bootstrap values (based on 1000 replications), only the values ≥50% are presented here. Scale bar depicts amino acid substitutions per site

The NJ and MJ based phylogenetic tree topologies of the FZD family reveal two major clusters. Cluster-I is further divided into three sub-clusters; sub-cluster of *FZD10, FZD9* and *FZD4,* sub-cluster of *FZD5-FZD8*, and sub-cluster of *FZD7, FZD2* and *FZD1*. Cluster-II consists of *FZD3-FZD6* (Fig. 1; Additional file 1). The tree topology pattern further suggests that Frizzled receptor family is diversified by in total 9 duplication events (Fig. 1; Additional file 1). First duplication has occurred after the divergence of eumetazoa from metazoans separating the ancestral genes of Cluster-I from ancestor of Cluster-II (Fig. 1; Additional file 1). Second duplication event has occurred at least prior to the bilaterian-nonbilaterian split separating the ancestral gene of sub-cluster *FZD7, FZD2* and *FZD1* from the ancestral genes of sub-clusters *FZD10, FZD9*, *FZD4, FZD5* and *FZD8* (Fig. 1; Additional file 1). The third duplication event results in the split-up of the ancestral genes of *FZD5* and *FZD8* from the ancestors of *FZD10, FZD9* and *FZD4* (Fig. 1; Additional file 1). This duplication event occurred at least prior to the divergence of protostomes and deuterostomes (Fig. 1; Additional file 1). Fourth duplication event has transpired at the root of Nephrozoa approximately 650 million years ago and is responsible for the separation of *FZD4* and ancestral gene of *FZD9/FZD10* (Fig. 1; Additional file 1)*.* The remaining five duplication events are specific to the vertebrate lineage (Fig. 1; Additional file 1). Furthermore, tree topology revealed that fifteen specie-specific duplications have occurred at different evolutionary time periods both in invertebrate and vertebrate FZD homologs (Fig. 1; Additional file 1).

### Domain organization of frizzled receptor family

**Fig. 2:**
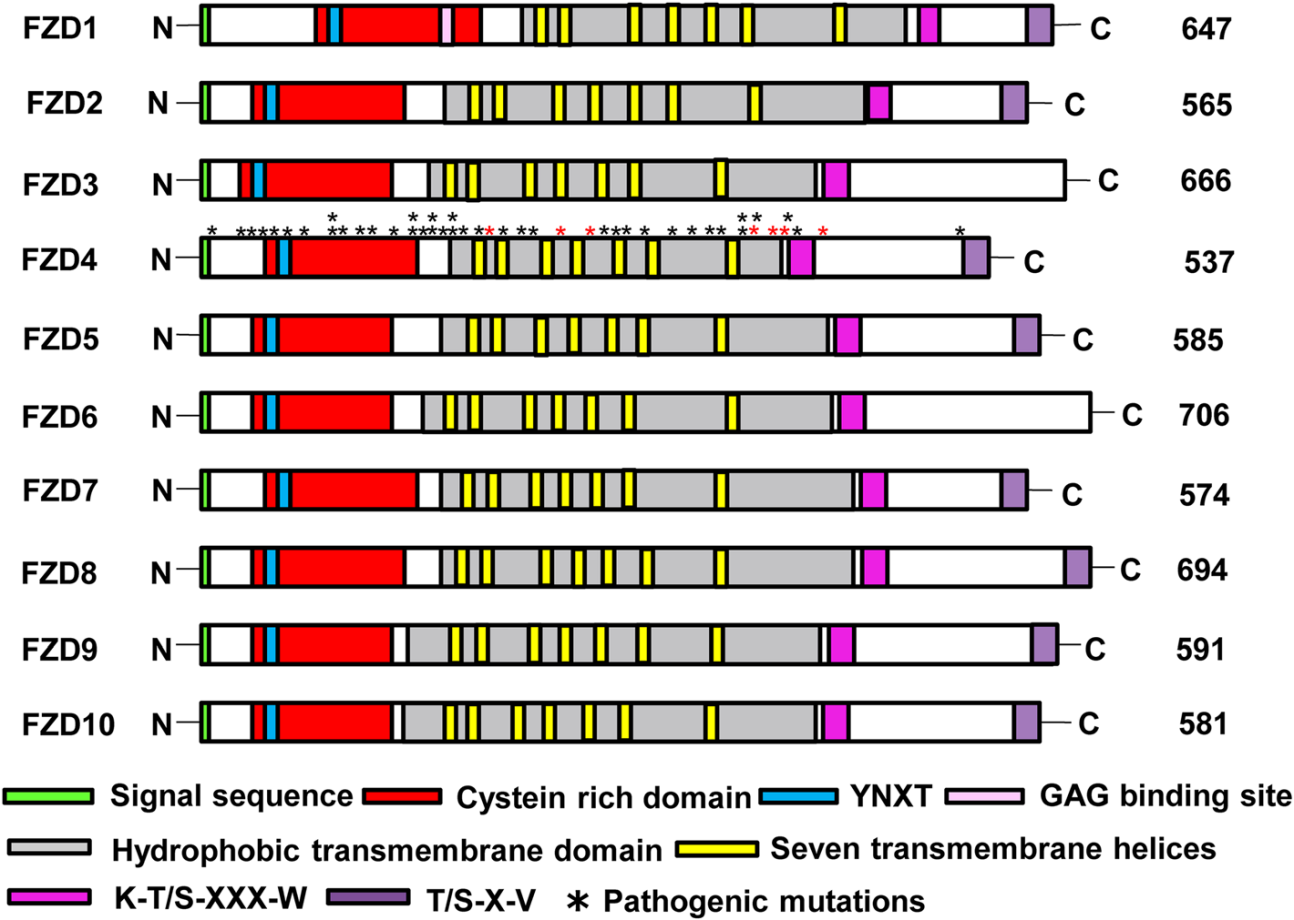
Domain organization of human Frizzled receptors. Schematic illustration depicts comparative organization of key functional domains and motifs of Frizzled receptor across human paralogs. Protein lengths are drawn approximately to scale. Domains and motifs are color coded. Asterisk symbol indicates the positions of 47 different FEVR causing variants on human FZD4 protein (40 missense variants are indicated with the black asterisk symbol, whereas 7 nonsense variants are depicted by red asterisks)

In order to gain insight into comparative domain organization of frizzled receptor family, previously reported domains and motif of this family are searched through extensive literature survey and mapped on putative human paralogs (Fig. 2).

Comparative domain investigation exhibits an amino-terminal membrane localizing signal peptide sequence rich in hydrophobic residues (Fig. 2). This signal peptide is followed by a 120 residues long conserved Cystein Rich Domain (CRD) (Fig. 2). Amino-terminal Cystein Rich Domain (CRD) comprehends YNXT motif which is highly conserved among all the putative paralogs of FZD family (Fig. 2). This motif is located exactly 5 residues after the second cysteine residue of CRD domain and considered to be a potential N-glycosylation site. This site might play a significant role in Wnt ligand binding [15]. CRD domain is necessary for WNT-FZD interaction and activation of Wnt pathways (Fig. 2) [16]. In addition, FZD1 contains an extra Glycosaminoglycan (GAG) binding site RxR residing on position 194–196 of CRD domain (Fig. 2). Previous investigation suggests that CRD domain of FZD1 requires glycosaminoglycans (GAGs) for regulation of signal transduction and cell proliferation [16].

The transmembrane (TMs) domain is conserved among all the putative paralogs of the family (Fig. 2). The occurrence of this domain in frizzled receptors indicates that this family belongs to the superfamily of G- Protein Coupled Receptors (GPCRs). It is the membrane spanning region of frizzled receptors which is predicted to contain seven transmembrane alpha helices [17]. The carboxyl-terminal region contains conserved K-T/S-XXX-W PDZ-binding motif that is located immediately after the hydrophobic transmembrane domain (Fig. 2) [18–20]. This motif is conserved among all the members of FZD family and is very essential for initiation of canonical Wnt pathway and for phosphorylation of Disheveled (Dvl) protein [21]. Interaction between FZD-Dvl completely diminishes when this motif encounters any mutation [21].

The comparative domain investigation revealed another carboxy-terminal residing T/S-X-V PDZ binding motif, present in all the members of FZD family except FZD3 and FZD6 (Fig. 2) [21]. This motif resides exact 14 residues downstream of internal Dvl-PDZ binding motif in FZD1, FZD2 and FZD7, while this gap is greater than 29 residues in other FZD family members (Fig. 2).

### Structural analysis of wild-type and mutant FZD4

**Fig. 3:**
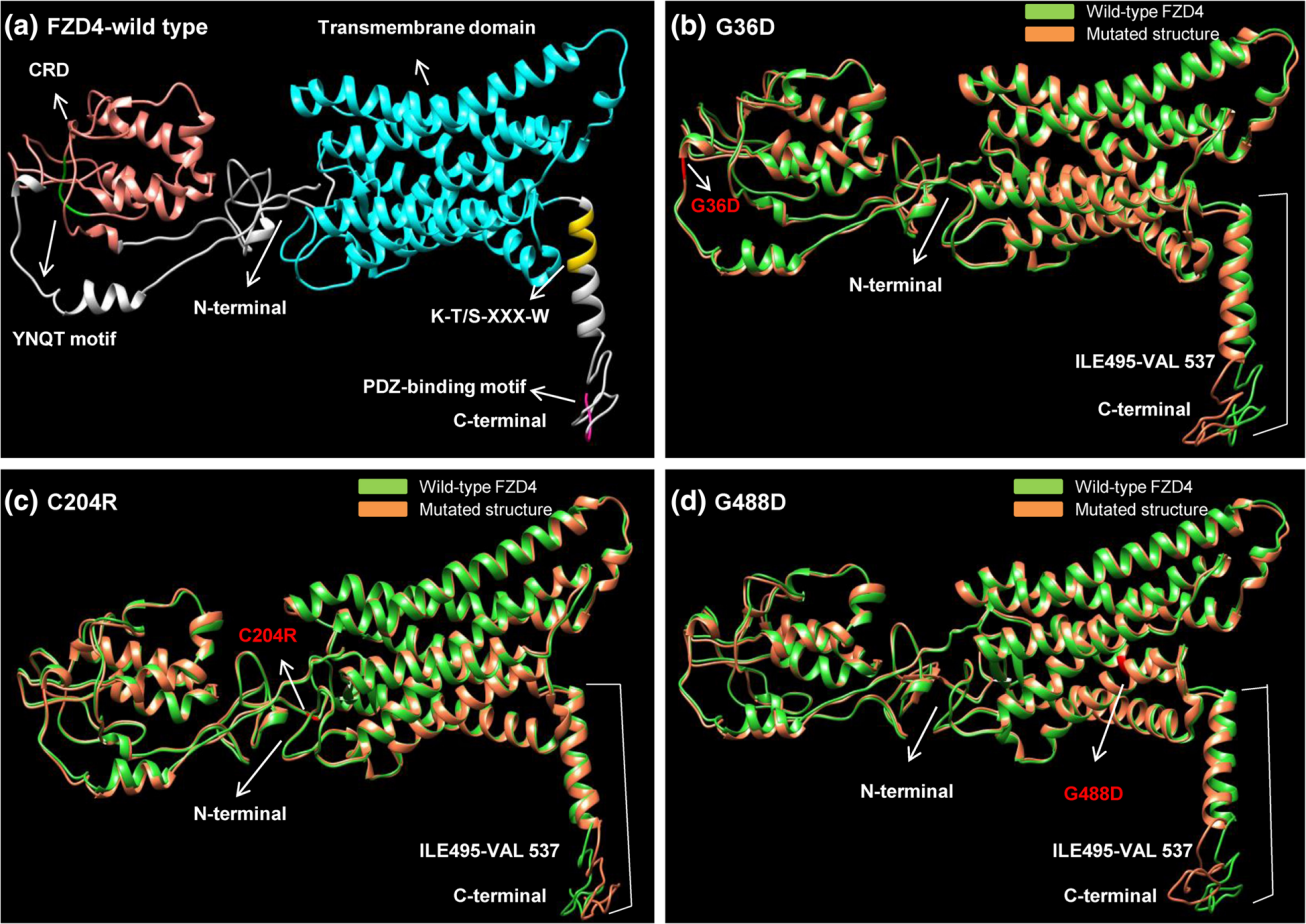
Structural deviations between human wild type and mutated FZD4 structures. Major structural shifts caused by disease associated missense mutations of the FZD4 protein were observed in the K-S/T-XXX-W and T/S-X-V PDZ binding motifs present in the carboxyl-terminal region. Mutated residues are labeled in red. **a** Represents the structure of wild type FZD4 protein in which all domains and motifs are color coded. **b** Shows structural superposition of wild type FZD4 (green) and mutated model G36D (coral peach). **c** Depicts structural comparison between wild type FZD4 (green) and mutated model C204R (coral peach). **d** Represents the structural deviations among wild type FZD4 (green) and mutated model G488D (coral peach)

In order to study the impact of mutations causing FEVR on overall conformation of FZD4 protein, a comparative structural analysis was performed (Fig. 3; Additional file 2). The full length X-ray crystallography structure of FZD4 is not available, therefore, three dimensional structure of wild-type human FZD4 protein is predicted through the I-TASSER structure prediction server [22]. The predicted wild-type protein structure is taken as a reference and compared with 40 predicted mutated models (previously reported FEVR causing FZD4 missense mutants). The predicted structures of wild-type and mutated proteins show high scores when their quality is evaluated through RamPAGE and ERRAT (Additional file 3) [23]. Structural deviations are evaluated with RMSD values [24]. All of the superimposed models (wild-type and 40 mutated structures) expose a common deviated region (495–537) (Fig. 3; Table 1; Additional file 2). This region possesses the K-T/S-XXX-W Dvl-PDZ binding motif and the T/S-X-V PDZ recognition motif at the carboxyl-terminal [9] (Table 1). Therefore, this particular region (amino acids; 495–537) of FZD4 protein is considered as critical with respect to its structure, function and involvement in FEVR. In addition to C-terminal deviated residues (495–537) shared among all mutants, structural deviations are also detected in highly conserved N-terminal extracellular cysteine-rich domain (CRD) and seven transmembrane domains of FZD4 (Fig. 3; Table 1; Additional file 2).

**Table 1:** Structural deviation in the backbone torsion angles of the wild type and mutated human FZD4 proteins

Comparison of wild-type and mutated structures	Major change in backbone torsion angles (residue number)	Major shift in regions	Critical region
P33S	44,45,110,132-135,137,141-143,147–150	CRD domain	
	285,417–432	Seven TMs domain.	
	496–537	K-T-XXX-W, PDZ binding motif	
G36D	41–46,70,71,133-138,141–150	CRD domain	
	416–431,468	Seven TMs domain.	
	495–537	K-T-XXX-W, PDZ binding motif	
E40Q	135–144,156–158	CRD domain	
	182–186,199, 202	UCR	
	243–244,248,284-286,337-338,417-420,466–469	Seven TMs domain	
	505–537	PDZ binding motif	
Y58C	134–135,141-144,147–150	CRD domain	
	243–244,248,284-285,334-337,416-431,467-469,495–497	Seven TMs domain	
	508–537	PDZ binding motif	
H69Y	147–149	CRD domain	
	285,287,334-339,415–432	Seven TMs domain	
	504–537	K-T-XXX-W, PDZ binding motif	
M105 T	274–278,284-286,467,468	Seven TMs domain	
	506–537	PDZ binding motif	495–537
M105 V	107–114,132–135	CRD domain	
	284–286,332-338,416-430,467–469.	Seven TMs domain	
	505–537	PDZ binding motif	
I114T	15–44,49,62-81,110,118,134–156	Signal peptide and CRD domain	
	213–214,274–275	Seven TMs domain	
	495–537	K-T-XXX-W, PDZ binding motif	
C117R	49,70-71,73–74	CRD domain	
	274–275	Seven TMs domain	
	496–537	K-T-XXX-W, PDZ binding motif	
R127H	15–44,49,63-81,110-111,118,133–156	Signal peptide and CRD domain	
	173–179	UCR	
	213–214,274–275	Seven TMs domain	
	496–537	K-T-XXX-W, PDZ binding motif	
M157 V	133–135,142–151	CRD domain	
	284,285,337,338,417-423,428-431,467–470	Seven TMs domain	
	496–537	K-T-XXX-W, PDZ binding motif	
C181R	419–422	Seven TMs domain	
	503–537	PDZ binding motif	
C181Y	420–423	Seven TMs domain	
	508–537	PDZ binding motif	
C204R	69–72,134,135,144–150	CRD domain	
	337,338,418-420,430,431	Seven TMs domain	
	495–537	K-T-XXX-W, PDZ binding motif	
C204Y	66–67,70-71,102,141–150	CRD domain	
	243–244,419–420	Seven TMs domain	
	504–537	K-T-XXX-W, PDZ binding motif	
Y211H	496–537	K-T-XXX-W, PDZ binding motif	
M223K	504–537	K-T-XXX-W, PDZ binding motif	
T237R	214,274–275	Seven TMs domain	
	503–537	K-T-XXX-W, PDZ binding motif	
R253C	504–537	K-T-XXX-W, PDZ binding motif	
W335C	274–275	Seven TMs domain	
	496–537	K-T-XXX-W, PDZ binding motif	
A370G	46–52,68-80,141	CRD domain	
	504–537	K-T-XXX-W, PDZ binding motif	
R417Q	42–46,147-150,134-137,147–150	CRD domain	
	418–432	Seven TMs domain	
	507–537	PDZ binding motif	
G488D	41–46,109-114,133–151	CRD domain	
	416–432	Seven TMs domain	
	495–537	K-T-XXX-W, PDZ binding motif	
G488 V	38,70	CRD domain	
	505–537	PDZ binding motif	
S497F	70–71,73–74	CRD domain	
	503–537	K-T-XXX-W, PDZ binding motif	
K499E	147–148	CRD domain	
	284–286, 336–338,418–431, 468–470	Seven TMs domain	
	505–537	K-T-XXX-W, PDZ binding motif	

This table shows the impact of FEVR associated missense mutations in the FZD4 protein on its backbone torsion angles by comparing them with its normal structure. The critical region encompasses the K-T-XXX-W and T/S-X-VPDZ binding motifs. In the first column, amino acid residue on the left indicates the wild-type residue; the number shows the amino acid position of the residue in the protein sequence, while the residue on the right shows the mutated residue. The second column specifies the positions at which major structural deviations were observed

The third column depicts the deviated region/residues shared among all mutant proteins analyzed (critical region)

*CRD* cysteine rich domain, *TM* transmembrane, *UCR* uncharacterized region

## Discussion

Increasing availability of the genome sequence data and high throughput annotation of genes permitted the molecular evolutionary analysis of various human genes and gene families [25]. Frizzled receptor family encode proteins that constitute the key component of Wnt signaling pathway with numerous developmental roles including cell proliferation, cell differentiation, tissue hemostasis and cell apoptosis [4]. The present study is an attempt to relate the evolutionary history and comparative structural aspects of human Frizzled receptors with particular phenotypic trait and disease.

NJ and ML based phylogenies of the FZD family (Fig. 1; Additional file 1) supported by high bootstrap scores revealed the complex evolutionary relationship among members of Frizzled receptor family. The phylogenetic tree topology portrayed a very ancient evolutionary history of Frizzled receptor family. The overall branching pattern revealed that FZD3 and FZD6 are the most ancient members of this family and have evolved prior to the divergence of placozoa (trichoplax) from eumetazoans, forming the most basal branch. The remaining members of this family are diversified in ParaHoxozoa history prior to tetrapod-teleost split. The close phylogenetic relationship among gene family members might depict their functional resemblance and similar cellular localization [26, 27]. For instance, both FZD3 and FZD6 are expressed in central nervous system (CNS) and are known to participate in the neural tube closure-related planer cell polarity (PCP) pathway and axonal growth during development [28]. In addition, FZD1, FZD2, and FZD7 falling in the same clade also share their functions in closure of the palate and ventricular septum [26].

Inspecting the protein domain topologies of the putative paralogs revealed conserved domain features; a cysteine rich domain and a seven helix transmembrane domain. However, some cluster specific differences in motifs were observed. For example, in the carboxyl-terminal PDZ binding site, a three residue motif crucial for the binding of PDZ domain of the DVL proteins is present in all putative members of the family except FZD3 and FZD6 [29]. The most divergent positioning of FZD3 and FZD6 in the phylogenetic tree and the absence of the C-terminal PDZ binding motif in these genes suggests that this short motif might have originated at the root of ParaHoxozoa after first duplication event approximately 680 million years ago (Figs. 1 and 2). Comparative domain analysis has further revealed that the amino-terminal region of this family is more preserved than the carboxyl-terminal region. This might reflect fundamental biological role and strong purifying selection on amino-terminal regions of Frizzled proteins. For instance, the N-terminal extracellular CRD domain determines binding specificity for Wnt ligands and transduces Frizzled-mediated Wnt/*β*-catenin signaling pathway [30]. The least preserved COOH-tail of Frizzled family members is known to be inherently unstructured [31]. Such disordered regions of a given protein are expected to evolve more rapidly and exhibit low level of sequence conservation than structured regions in the same protein because they are not constrained by structure [32].

*FZD4* gene is well understood among all members of the family in terms of its biological role and involvement in disease, therefore we sought to infer the protein structural basis for familial exudative vitreoretinopathy (FEVR) phenotype caused by mutations in this gene. Comparative structural analysis of human wild-type FZD4 and its mutant structures revealed that even though the FEVR-causing missense changes scattered across the FZD4 protein structure, all of them appears to impact the confirmation of the C-terminal 495–537 region through a phenomenon called “epistasis” (Fig. 3; Table 1) [33]. The functional significance of this critical region is supported by previous data, as it harbors two functionally crucial binding sites [29]. For instance, residues 499–505 of critical region exhibits the internal cytoplasmic K-T/S-XXX-W motif that directly interacts with the PDZ domain of Dvl (Dishevelled) [34], whereas residues 535–537 of critical region contain distal C-terminal S/T-X-V PDZ-recognition motif that also interacts with the PDZ domain bearing proteins such as post-synaptic density protein (PSD) and GRIP1 [35]. The C-terminal of FZD4 is an intrinsically disordered region and therefore possesses structural plasticity [31]. Such inherently disordered region of proteins are capable of adopting a variety of structurally unrelated conformations whose features/functions are largely dependent on the cellular environment and/or on the available interacting partners [32, 36]. The effect of structural deviations in response to FEVR causing missense mutation could be deleterious either because it destabilizes structure of FZD4 or alters its intracellular localization or disrupt ligand-binding and interaction with other proteins. For instance, amongst the identified missense variants associated with FEVR; a phenylalanine-to-serine mutation at amino acid position 328 (F328S) of intracellular loop 2 (iloop2) yields a FZD4 with a reduced capability to activate the Tcf/Lef transcription in response to Norrin. Intriguingly, this FZD4 mutant was unable to employ Dvl2 to the cell membrane through C-terminus KTXXXW motif at the transmembrane domain 7 proximal segment of the cytosolic tail. Based on these biochemical data it can be suggested that iloop2 of FZD4 may modulate interactions between Frizzled and other signaling molecules through distantly located carboxy-terminus region [37]. Furthermore, a deletion of two nucleotides that led to a frameshift and created a stop codon at amino-acid 533 (L501fsX533) resulted in abnormal PDZ binding motif at C-terminus of FZD4 and hence leading to FEVR [31]. Biochemical investigations show that this particular frameshift mutation (L501fsX533) generates a disorder-to-order structural transition in the C-terminal cytosolic tail of FZD4. The resulting mutant FZD4 protein fails to reach the Plasma Membrane of the cells and accumulates in the Endoplasmic Reticulum [31]. These previously reported functional data supports the prediction that the critical region (amino acid positions 495-537at the C-terminus) of FZD4 protein identified in the present study is crucial for normal function and any conformational change in this region contributes to the disease pathology. Comparative structural analysis also revealed structural deviation in cysteine-rich domain (CRD) and seven transmembrane domains of subset of FZD4 mutant proteins analyzed. In particular, the N-terminal extracellular CRD domain is conserved among Frizzled family members and determines binding specificity for Wnt ligands [30]. For instance, the extracellular ligand Norrin binds specifically to the CRD domain of FZD4 but not the CRDs of other members of mammalian Frizzleds [38]. This FZD4-Norrin binding results in Wnt/β-catenin pathway activation in normal retinal vascular development [30, 38]. Missense mutations within and near the highly conserved CRD domain are known to affect correct protein folding of the CRD and disrupt Norrin binding, consequently, causing FEVR [30]. Therefore, the protein conformational changes in CRD domain of FZD4 mutants can potentially affect retinal development and contributes to disease pathogenesis.

## Conclusion

Frizzled receptor family is an evolutionary conserved multigene family with a fundamental biological role in metazoans development. The phylogenetic data suggests the origin of Frizzled receptor family in early metazoans and subsequently diversified at different time points during bilaterians history. The evolutionary investigations reveal that Frizzled receptors have undergone extensive functional diversification through complex pattern of gene duplications and sequence divergence. Comparative structural analysis of the FZD4 protein pinpoints a critical region (amino acids 495–537) with crucial implications in normal cellular function and disease pathogenesis (FEVR). These findings provide a base for future structural and evolutionary studies to elucidate further the role of Frizzled receptors in metazoans evolution, development and disease.

## Methods

Members of the Frizzled receptor family and their human protein sequences were identified and extracted from Ensembl Genome Browser [39]. In total, 59 putative orthologous protein sequences were retrieved from Ensemble Genome Browser and National Center for Biotechnology Information (NCBI) by using BLASTp bidirectional searches [40, 41]. Further confirmation of the common ancestry of the putative orthologs was obtained by clustering homologous proteins within phylogenetic trees. Sequences whose position within a tree was in sharp conflict with the uncontested animal phylogeny were excluded from the analysis.

The species that were selected for sequence analysis includes *Homo sapiens* (human), *Callithrix jacchus* (marmoset), *Mus musculus* (mouse), *Rattus norvegicus* (rat), *Equus caballus* (horse), *Monodelphis domestica* (opossum), *Gallus gallus* (chicken), *Anolis carolinensis* (anole lizard), *Danio rerio* (zebrafish), *Takifugu rubripes* (fugu), *Tetraodon nigroviridis* (tetraodon), *Gasterosteus aculeatus* (stickleback), *Oryzias latipes* (medaka), *Lepisosteus oculatus* (spotted gar), *Petromyzon marinus* (lamprey), *Latimeria chalumnae* (coelacanth), *Ciona intestinalis* (C.intestinalis), *Ciona savignyi* (C.savignyi), *Drosophila melanogaster* (fruitfly), *Caenorhabditis elegans* (*C. elegans*), *Capitella teleta* (capitella), *Helobdella robusta* (Californian leech), *Octopus bimaculoides* (octopus), *Nematostella vectensis* (starlet sea anemone), *Trichoplax adhaerens* (trichoplax).

### Sequence analysis

Phylogenetic analysis of Frizzled receptor family was performed using Neighbor-joining method (NJ) [42]. Uncorrected p-distance and Poisson corrected (PC) amino acid distance were used as amino acid substitution models [43]. Both models produced similar results, therefore, only NJ tree based on uncorrected p-distance is presented here (Fig. 1). Maximum likelihood (ML) tree based on Whelen and Golman (WAG) amino acid substitution model was also constructed (Additional file 1) [44, 45]. To test the reliability and correctness of NJ and ML trees, bootstrap method (at 1000 pseudo replicates) was used which produced the bootstrap value for each internal branch in trees [46].

Comparative protein domain analysis was conducted to assign domains, motifs and sub-motifs to each member of Frizzled receptor family [20, 29, 47, 48]. The positions of domains and motifs on each putative paralogs were identified and mapped by employing a multiple sequence alignment tool, the Clustal Omega [49]. Approximate scaling and positioning of the respective domains and motifs was also carried out. Previously reported disease causing mutations in FZD family members were also mapped.

### Structural analysis

To date, approximately 47 different FEVR pathogenic mutations have been associated with human *FZD4*, of which ~ 40 are missense variants dispersed across the protein structure [10, 11]. These 40 missense mutations are targeted for protein structural and conformation analyses (G22E, P33S, G36D, E40Q, C45Y, Y58C, H69Y, M105 T, M105 V, C106G, I114T, C117R, R127H, M157 V, M157K, E180K, C181R, C181Y, K203 N, C204R, C204Y, Y211H, M223K, T237R, R253C, I256V, W335C, A339T, M342 V, A370G, R417Q, T445P, R466W, D470N G488D, G488 V, G492R, S497F, K499E, G525R) [50–52]. For this purpose, the structure of wild-type FZD4 protein sequence predicted through I-TASSER and taken as a reference model to study the impact of selected subset of missense mutations on overall conformation of protein [22]. The mutant structures of FZD4 are predicted through MODELLER (version 9.14) [53]. Best structures are selected on the basis of Discrete Optimized Protein Energy (DOPE) score. Mutant models are superimposed over wild-type FZD4 by using Chimera and Root Mean Square Deviation (RMSD) values are evaluated [24, 54]. All forty mutant protein structures have RMSD values > 0.4 Å. The effect of these mutations on protein stability was also investigated through MuPro [55]. The overall model quality of the predicted proteins are analyzed through structure validation tools ERRAT and Ramachandran plot [23, 56].

## Additional files

Additional file 1: Phylogenetic analysis of frizzled receptors gene family. (PDF 505 kb)

Additional file 2: Structural comparison of wild type and Familial Exudative Vitreoretinopathy mutated FZD4. (PDF 1958 kb)

Additional file 3: Evaluation of 3D models of mutant FZD4 proteins. (PDF 6199 kb)

## Abbreviations

CRD: Cystein Rich Domain
DOPE: Discrete Optimized Protein Energy
Dvl: Disheveled
FEVR: Familial exudative vitreoretinopathy
FZDs: Frizzleds
GAG: Glycosaminoglycan
GPCRs: G-protein coupled receptors
ML: Maximum Likelihood
NCBI: National Center for Biotechnology Information
NJ: Neighbor-joining
PC: Poisson corrected
PCP: Planer cell polarity
RMSD: Root Mean Square Deviation
TM: Transmembrane
WAG: Whelan and Goldman
