# Supplementary material for: Molecular evolutionary and structural analysis of familial exudative vitreoretinopathy associated FZD4 gene
Source: BMC Evol Biol. 2019 Mar 8;19:72. doi: 10.1186/s12862-019-1400-9 (PMC6408821; doi:10.1186/s12862-019-1400-9)
Supplement: Supplementary file 1 — Phylogenetic analysis of frizzled receptors gene family. (PDF 505 kb) [file 12862_2019_1400_MOESM1_ESM.pdf]

Supplementary Fig. 1

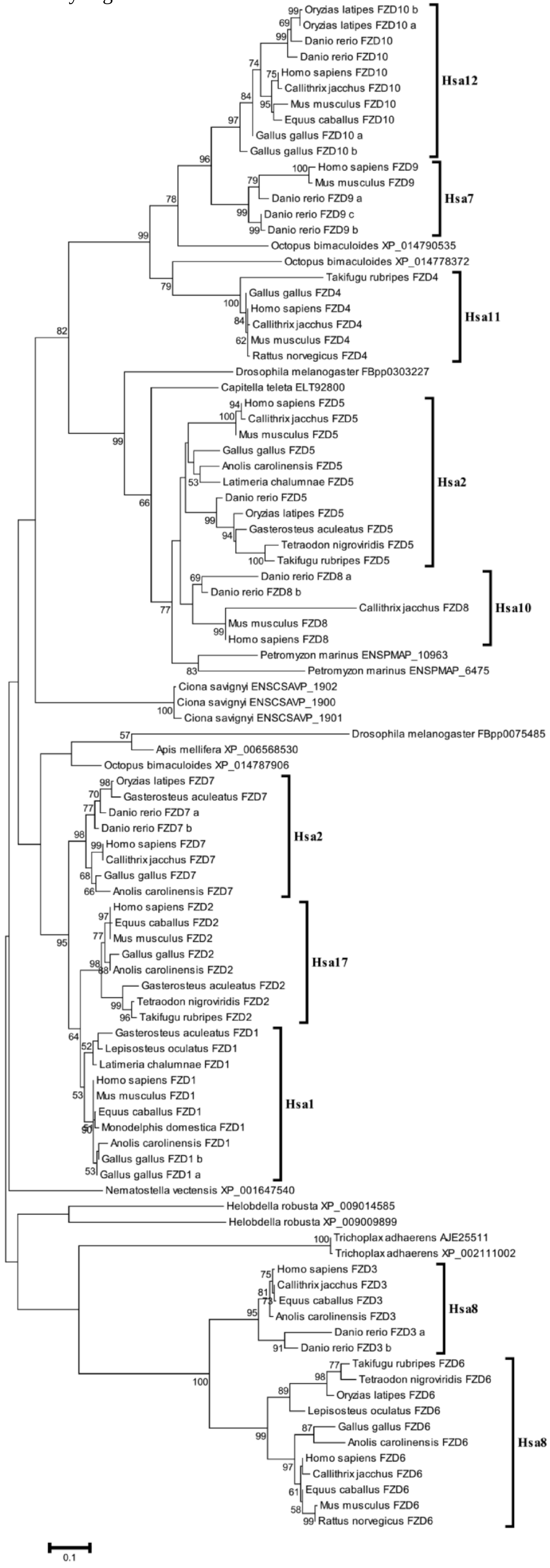

Supplementary Fig. 1 Phylogenetic analysis of frizzled receptors gene family. The phylogenetic relationship among frizzled receptors genes was inferred by Maximum Likelihood method through Whelan And Goldman model. This analysis involved 97 amino acid sequences of 10 frizzled receptor genes. The tree is drawn to scale, with branch lengths measured in the number of substitutions per site. There were a total of 237 positions in the final dataset after eradicated all positions containing gap and missing data
