# Supplementary material for: Molecular evolutionary and structural analysis of familial exudative vitreoretinopathy associated FZD4 gene
Source: BMC Evol Biol. 2019 Mar 8;19:72. doi: 10.1186/s12862-019-1400-9 (PMC6408821; doi:10.1186/s12862-019-1400-9)
Supplement: Supplementary file 2 — Structural comparison of wild type and Familial Exudative Vitreoretinopathy mutated FZD4. (PDF 1958 kb) [file 12862_2019_1400_MOESM2_ESM.pdf]

Supplementary Fig. 2

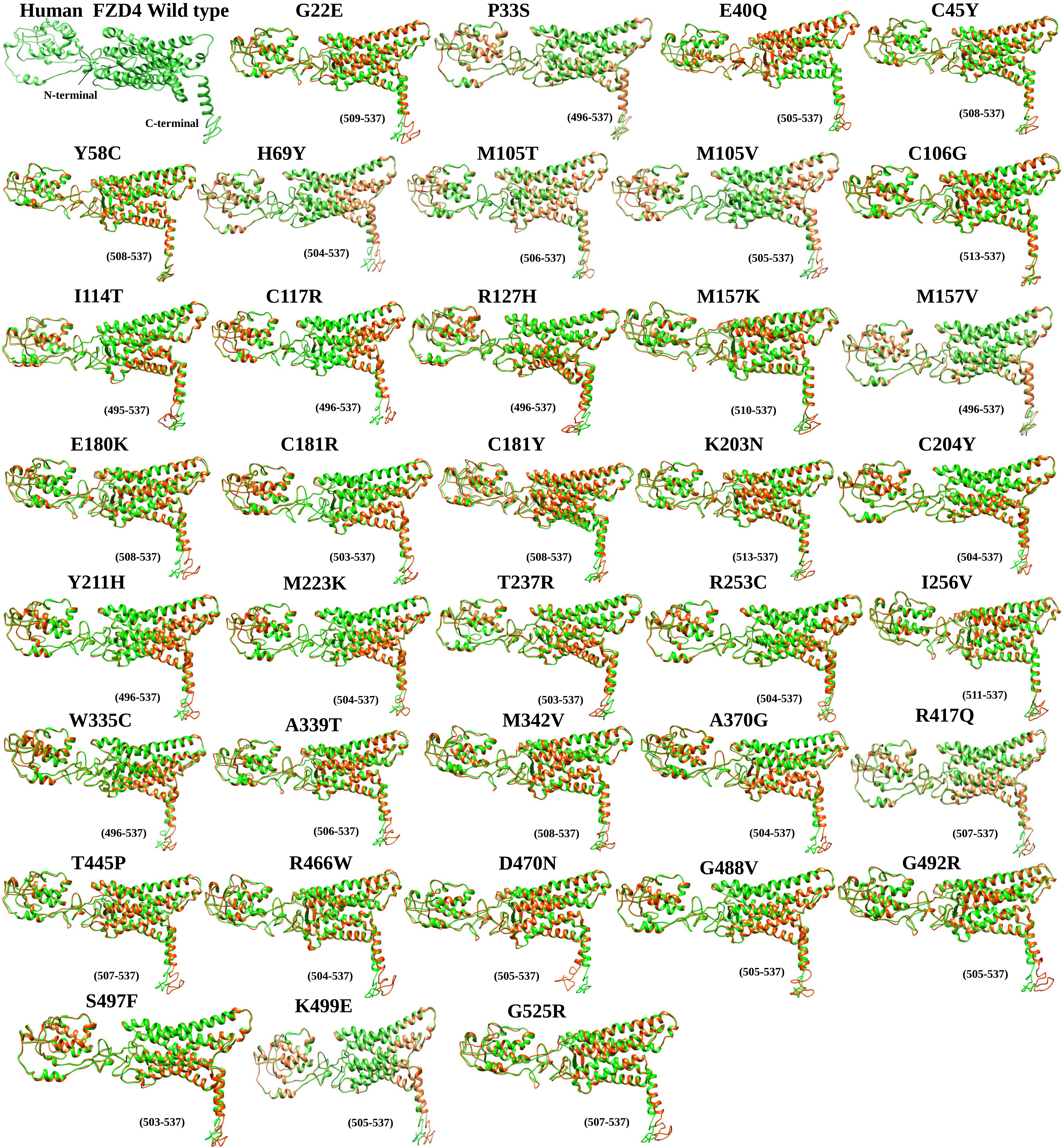

Supplementary Fig. 2 Structural comparison of wild type and Familial Exudative Vitreoretinopathy mutated FZD4. This figure shows the superposition of each mutant structure (coral peach) over wild type human FZD4 (green) The numbers in the parenthesis depicts the major deviated structural region in the mutated structures
