# Supplementary material for: Molecular evolutionary and structural analysis of familial exudative vitreoretinopathy associated FZD4 gene
Source: BMC Evol Biol. 2019 Mar 8;19:72. doi: 10.1186/s12862-019-1400-9 (PMC6408821; doi:10.1186/s12862-019-1400-9)
Supplement: Supplementary file 3 — Evaluation of 3D models of mutant FZD4 proteins. (PDF 6199 kb) [file 12862_2019_1400_MOESM3_ESM.pdf]

Supplementary Fig. 3

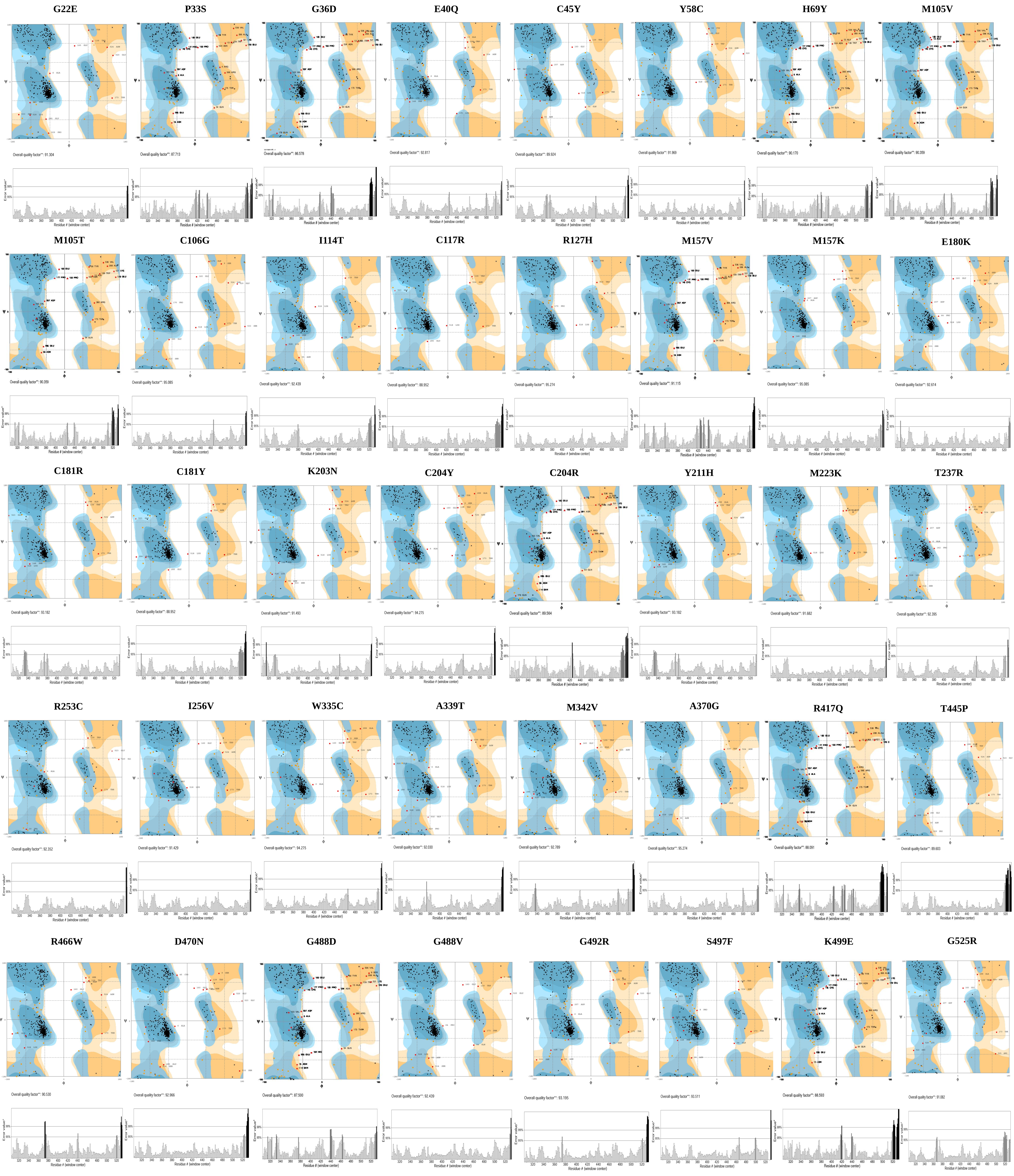

Supplementary Fig. 3 Evaluation of 3D models of mutant FZD4 proteins. Rows 1 and 3 depicts Ramachandran plots of mutated human FZD4 protein involved in Familial Exudative Vitreoretinopathy. These plots elucidate the quality of the models in terms of presence of the residues in fallowed/disallowed region While rows 2 and 4 Shows results of ERRAT, describes the overall quality factor of the models. Overall quality factor is expressed as percentage of the protein for which the calculated error value falls below the 95% rejection limit, calculated by Errat
